# Molecular Characterization of Plasmid pMbo4.6 of *Moraxella bovis* ATCC 10900

**DOI:** 10.1007/s00284-012-0257-6

**Published:** 2012-11-06

**Authors:** Beata Furmanek-Blaszk, Natalia Kurpiewska, Robert Boratynski, Marian Sektas

**Affiliations:** Department of Microbiology, University of Gdansk, Kladki 24, 80-822 Gdansk, Poland

## Abstract

We report the characterization of a small cryptic plasmid unlike any previously described from *Moraxella bovis* ATCC 10900, a Gram-negative bacterium belonging to the family Moraxellaceae. The complete nucleotide sequence of the plasmid pMbo4.6 was determined. The plasmid was analyzed and found to be 4658 in size with a G+C content of 38.6 mol %. Computer analysis of the sequence data revealed four major open reading frames encoding putative proteins of 10.1 (ORF1), 64.2 (ORF2), 45.7 (ORF3), and 12.1 kDa (ORF4). ORF1 and ORF2 encode proteins that show a high level of amino acid sequence similarity (44 %) with some mobilization proteins. ORF3 encodes a protein showing a relatively high amino acid sequence similarity (about 40 %) with several plasmid replication initiator proteins. Upstream of ORF3, a 320-bp intergenic region, constituting the putative origin of replication that contained an AT-rich region followed by four direct repeats, was identified. This set of repeated sequences resembles iteron structures and plays an important role in the control of plasmid replication by providing a target site for the initiation of transcription and replication factors (IHF and RepA). Several palindromic sequences, inverted repeats, and hairpin-loop structures, which might confer regulatory effects on the replication of the plasmid, were also noted. ORF4 encodes an uncharacterized protein, conserved in bacteria, belonging to the DUF497 family. Sequence analysis and structural features indicate that pMbo4.6 replicates by a theta mechanism.

## Introduction

*Moraxella bovis* is the bacterial agent most frequently isolated in acute and chronic cases of infectious bovine keratoconjunctivitis (IBK) [17]. This organism is rod-shaped, Gram-negative, oxidase-positive and nonmotile. The ability to cause IBK is associated with the production of a certain virulence factor. Plasmids may specify a variety of factors that enhance survival or virulence in pathogenic bacteria. The factors mediated by these plasmids include antibiotic resistance, adhesions, hemolysins and exotoxins. The majority of *M. bovis* strains are known to contain multiple plasmids ranging from 4 to 45 kb in size, but no relationships between the number of plasmids and virulence has been found [23, 28]. In fact, there have only been a few previous reports of plasmids being isolated from *M. bovis* and only one plasmid (pMBO-1) has currently been characterized. In 2006 Kakuda et al. [20] showed that *M. bovis* Epp63 contains a 44.2-kb plasmid pMBO-1 carrying two large ORFs, *flpA* and *flpB*, encoding proteins with similarity to *Bordetella pertusis* filamentous haemagglutinin. This strain also contains a 27-kb plasmid pMBO-2 which encodes several proteins involved in the conjugational transfer of plasmids. The recent discovery and determination of a complete sequence of conjugative and/or mobilizable plasmids and their possible ability for cell-to-cell transmission might be another important factor for the adaptive potential apparent in this group of bacteria [20, 34]. It was shown that the large plasmids share some regions of structural similarities indicating possible recombinational changes between them [20, 35]. However, there is a strong genetic barrier to interspecies transformation in the moraxellae [19].

This paper presents the complete nucleotide sequence of the pMbo4.6 plasmid of *M. bovis* ATCC 10900 and genetic analysis of its elements.

## Materials and Methods

### Bacterial Strains and Growth Conditions

The *M. bovis* strain ATCC 10900, generously provided by Dr. E Falsen (University of Göteborg, Sweden), was grown routinely on brain–heart infusion agar (BHI), tryptic soy (TS) agar with 5 % sheep blood or TS broth (Graso, Poland) and incubated in air at 37 °C. The *Escherichia coli* strain DH5α [15] was used for a routine cloning procedure. The *E. coli* MC1061 strain [6] was used for the β-galactosidase expression measurement. The overproduction of the RepA protein was performed with *E. coli* BL21(DE3) [30]. The *E. coli* strains were routinely grown and maintained in LB medium at 37 °C with appropriate antibiotic selection. The genomic DNA of the *M. bovis* strains, IBA, and T412 were kindly provided by Dr. T. Kakuda (Kitasato University, Japan).

### DNA Manipulation Techniques

Plasmid DNA was extracted from *M. bovis* and *E. coli* strains by the alkaline lysis technique [4]. Other molecular biologic procedures were followed as described by Sambrook et al. [27]. All the enzymes were purchased from either Fermentas (Lithuania) or New England Biolabs (USA).

### Nucleotide Sequence Analysis

To determine the complete nucleotide sequence of pMbo4.6, the plasmid was digested with Xmn*I* and then cloned into the Eco*RV* site of the pBR322 vector [5]. The derived plasmid was then sequenced on both strands using an automated DNA sequencer (model ABIPRISM 310 Genetic Analyzer; PE Applied Biosystems). Comparison searches through the databases were performed by the BLAST program [1] provided by the National Center for Biotechnology Information. The complete sequence of the plasmid was deposited in GenBank at accession no. GQ998872.

### Expression and Purification of the Recombinant Protein

The pMbo4.6 RepA coding region was amplified from the genomic DNA of *M. bovis* using the primer pair repATG with repTAA (Table 1) containing the Nde*I* or Eco*RI* restriction sites, respectively. The PCR product was cloned into pET24a that had been digested with Nde*I* and Eco*RI*, yielding plasmid pETrepA. pET24a is a vector that employs a phage T7 promoter for the expression of cloned genes. The integrity of pETrepA was verified by DNA sequencing before being transformed into *E. coli* BL21(DE3). From a 500 ml culture, cells were harvested, resuspended in 20 ml of buffer S (10 mM potassium phosphate pH 6.5, 20 mM KCl, 1 mM EDTA, 10 mM 2-mercaptoethanol, 5 % (v/v) glycerol) supplemented with a protease inhibitor 0.1 mM phenyl-methyl-sulfonyl-fluoride and disrupted by sonication at 4 °C in 60 × 10-s bursts. After centrifugation, the supernatant was applied to a 3.5 × 2.8 cm phosphocellulose P11 column equilibrated with buffer S. The column was then washed with 200 ml of buffer P and the proteins eluted with a 150 ml KCl gradient (20–1000 mM) in the same buffer. Fractions of 2 ml were collected and assayed for RepA protein's presence by 10 % SDS–polyacrylamide gel electrophoresis and Coomassie Blue staining.

**Table 1:** Primers

Primer	Sequence (5′–3′)^a^	Restriction enzyme
orirep1	CTAGGAGGGATTGACTACCAAA	
orirep2	CGCAAACCTTTGATACATAAGGC	
orirep3	AAGAATTCGGGCGTGGTTTTGGGGATTG	Eco*RI*
orirep4	GTGCTTTTTCGGTTTTGGCG	
orirep5	TTGGATCCTACAATTAAATCTTTGCTC	Bam*HI*
orirep6	CGCCAAAACCGAAAAAGCAC	
repATG	GACCTACATATGAGCAAAGATTTAATTG	Nde*I*
repTAA	TAAGAATTCCATTAGATATCCAAAA	Eco*RI*
prepG	AATTCATTTACAATAACTCTTTTTACTCGTATAATTAATTAG	
prepD	GATCCTAATTAATTATACGAGTAAAAAGAGTTATTGTAAATG	
megaG	AATTCTAGGAGTATTTACAATAACTCTTTTTAGGTGTATAATTAATTACTCCTTTTTTG	
megaD	GATCCAAAAAAGGAGTAATTAATTATACACCTAAAAAGAGTTATTGTAAATACTCCTAG	

^a^Nucleotides that are underlined denote engineered restriction endonuclease sites

### DNA-Binding Experiments

Primers were used directly in PCR amplification to generate probes which were purified by electroelution and stored at −20 °C. The orirep35 probe (nt 2381–2758), the orirep32 probe (nt. 2381–2564), and the orirep15 probe (nt 2573–2658) were generated with the primer pairs: orirep3/orirep5, orirep3/orirep2, orirep1/orirep5, respectively (Table 1). Electrophoretic mobility shift assays were performed by incubating DNA with increasing amounts of purified RepA protein in a 1 × binding buffer (10 mM Tris–HCl pH 7.5, 10 mM MgCl_2_, 100 mM NaCl, 0.2 mM dithiotreitol, and 5 % (v/v) glycerol) and a final reaction volume of 30 μl. After incubation at 22 °C for 20 min, the reaction mixtures were separated on 6 % polyacrylamide gels at 4 °C using a 0.4× TBE buffering system.

### Construction of the Plasmid for Promoter Analysis

To analyze the regulation of activity of the *repA* gene promoter, lacZ^+^ reporter plasmid pRS415 was used as the backbone [29]. For the cloning of the PCR-generated DNA fragment containing the *oriV* region (378 bp), including the promoter of the *repA* gene, and oligonucleotides orirep3 and orirep5 were used (Table 1), resulting in a pRSori35 plasmid. The second construct pRSplusIR was created as follows: the 59-bp synthetic DNA fragment formed after annealing the megaD and megaG oligonucleotides (Table 1), containing a predicted promoter region with three IR sequences within, was inserted upstream of the promoter-less reporter *lacZ* gene between the EcoRI and BamHI sites of the reporter vector pRS415. In addition to this, we cloned the non-iteron *repA* promoter region obtained by annealing the two oligonucleotides prepG and prepD (Table 1) and cloned it into the Eco*RI*–Bam*HI* digested vector pRS415. The constructed plasmid was designated pRSminusIR. All the resulting plasmids were introduced into the *E. coli* MC1061 cells containing the RepA-delivering plasmid pACYCaraRep in the L-arabinose inducible manner. This plasmid was constructed by subcloning the *araC*-*P*_araBAD_:*repA* DNA fragment from the pBAD24 derivative [14] into the Cla*I*–Hin*dIII* sites of pACYC177 [7].

### β-Galactosidase Assay

*E. coli* MC1061 cells bearing pRS415 derivatives and the RepA-delivery plasmid pACYCaraRep were grown at 37 °C in LB medium with aeration. The media were supplemented with the appropriate antibiotics and with l-arabinose for the induction of the *repA* gene expression, as indicated. The cells (0.1-ml culture aliquots) were permeabolized with 25 μl of 0.1 % sodium dodecyl sulfate and 50 μl of chloroform. Samples were equilibrated at 28 °C and assayed for a level of β-galactosidase produced from the fusion *lacZ* ^+^ gene. The reaction was started using 0.2 ml ONPG substrate (4 mg ml^−1^) until a yellow color developed, and then was stopped by the addition of 0.5 ml of 1 M Na_2_CO_3_. Units were determined by the following equation: OD_420_/OD_600_ × volume assayed (ml) × min, as described [24].

## Results

### Sequence Analysis of pMbo4.6

**Fig. 1:**
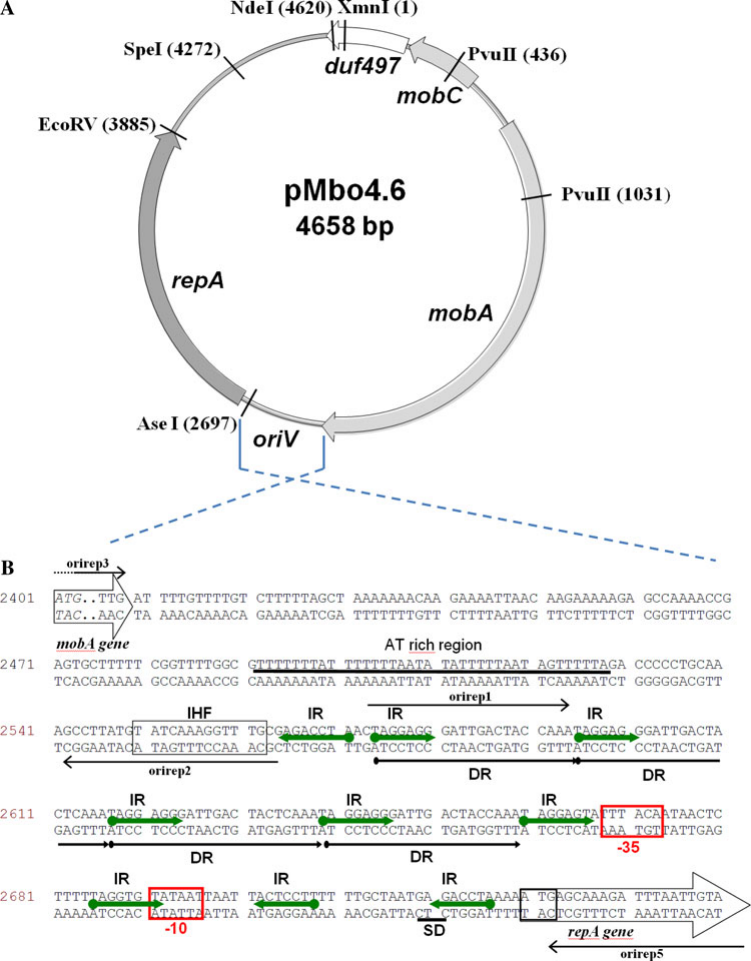

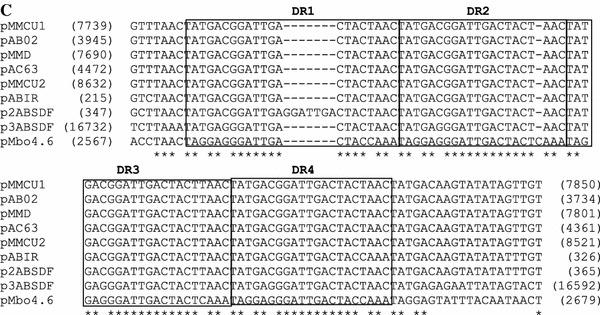
Genetic structure of pMbo4.6 plasmid. **a** Partial restriction map of the plasmid. The location of the mobilization proteins, the replication protein and the DUF497 protein are indicated. **b** Nucleotide sequence of the putative *oriV* region. The fragment includes the AT-reach region, IHF binding site, 4 directed repeats (DR, iterons;* solid lines* under sequence), 9 inverted repeats (IR, *arrows* between sequence strands), and −35 and −10 boxes of the *repA* gene promoter. *Thin black arrows* (*above and below*) represent the positions and orientations of the various primers (specified in Table 1) used in generating PCR products. Sequence numbering according to the pMbo4.6 sequence deposited at GenBank (Accession No. GQ998872). **c** Alignment of the *oriV* regions of the pMbo4.6 and p3ABSDF (CU468233), pABIR (EU294228), p2ABSDF (CU468232), pAC63 (JN982951), pMMD (GO904226, pMMCU2 (GQ476987), pMMCU1 (GQ342610), pAB02 (AY228470) *Acinetobacter* plasmids.* Asterisks* indicate identical nucleotides. The numbers at the* left* and* right* sides of each sequence refer to the deposited nucleotide sequence. Sequences were aligned by CLUSTAL W [31]

The plasmid pMbo4.6 isolated from *M. bovis* ATCC 10900 was subcloned and fully sequenced as follows. pMbo4.6 was linearized with Xmn*I* and cloned into the Eco*RV* site of pBR322 to construct pMbo9.2. The complete nucleotide sequences of both strands of pMbo4.6 were determined (GenBank Accession No. GQ998872). The G+C content of this plasmid was found to be 38.6 mol %, which is similar to those of plasmids pMBO-1 and pMBO-2 of *M. bovis* Epp63 [20]. Computer analysis of the sequence of pMbo4.6 revealed the presence of four open reading frames encoding putative proteins of 10.1 ORF1 (MobC), 64.2 ORF2 (MobA), 45.7 ORF3 (RepA), and 12.1 kDa ORF4 (DUF497), which together covered 73 % of the plasmid DNA. ORF2 and ORF3 were in the same transcriptional orientation and ORF1 and ORF4 in the reverse strand. A schematic representation of plasmid pMbo4.6 is shown in Fig. 1a.

To attribute functions to the deduced products of the ORFs, these were compared to the gene products available in the databases. Thus, ORF1 (the *mobC* gene) contains 276 bp and is found between coordinates 242 and 517. The putative promoter was recognized upstream of *mobC* (_578_TTTACG–17nt–TATAAT_550_) [16]. The MobC protein shares 46 and 44 % identity with the putative mobilization proteins encoded in plasmids pColE9-J (RefSeq Accession NC_011977) and pO26-S4 (NC_011228) from *E. coli*, respectively. The *mobA* gene which starts with an ATG codon is located between coordinates 733–2409 and encodes a protein of 558 amino acids with a predicted molecular weight of 64.2 kD. The deduced amino acid sequence exhibits a high level of identity with some plasmid mobilization proteins. The highest similarities between the translational product of ORF2 and other proteins were observed for the TraA protein of plasmid pSmeSM11a (GenBank Accession No. DQ145546) from *Sinorhizobium meliloti* and the Mob proteins of plasmids p2ABSDF (RefSeq Accession NC_010396) and p3ABSDF (NC_010398) from *Acinetobacter baumanii* (46 % identity). Both proteins belong to the MobA/MobL family known to be engaged in the conjugal transfer of plasmid DNA. A sequence comparison of the various Mob proteins showed that the highest similarities between these proteins were observed in their N-terminal parts. These conserved sequences correspond to the catalytic domain involved in DNA cleavage-joining activity. Sequence analysis of the protein encoded by the *mobA* gene reveals the presence of three conserved motifs which have been used to classify pMbo4.6 into the MOB_Q_ family relaxases (the catalytic Tyr in Motif I, conserved Glu in Motif II, and the three His in the 3H Motif III) [11].

Examination of the DNA sequence upstream of the *mobA* gene for possible promoter sequences reveals a 43-bp DNA fragment nearly identical (91 %) to the promoter region of the *mobC* gene associated with the mobilizable plasmid pEMCJH03 from *Moraxella catarrhalis* [18]. The DNA sequence between ORF1 and ORF2 may serve as the origin of the conjugal DNA transfer. There is a palindromic sequence with a 9-bp inverted repeat interrupted by the sequence CAA. This inverted repeat may be the structural elements that function as a recognition site for mobilization proteins. MobC acts together with MobA to form a complex called the relaxosome [11].

**Fig. 2:**
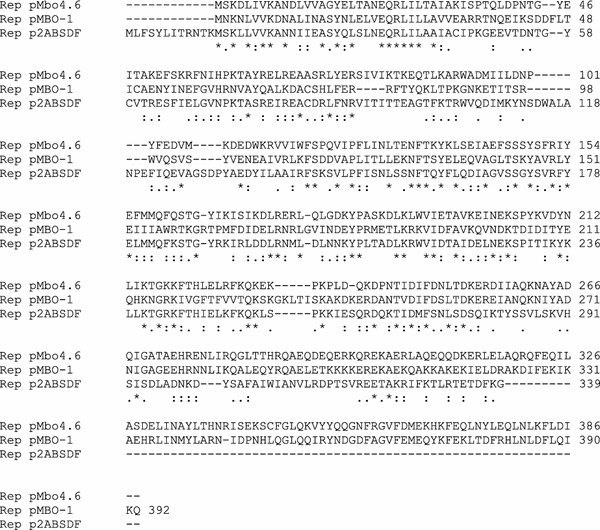
Comparison of the amino acid sequences of the Rep proteins. The numbers on the* right* indicate the amino acid position relative to the N-terminus. Identical residues are indicated by an* asterisk*;* two dots* denote a highly conservative substitution,* one dot* a conservative substitution. Sequences were aligned by CLUSTAL W [31]

The entire nucleotide sequence between coordinates 2730 and 3890 (ORF3) encodes a protein showing a relatively high amino acid sequence similarity with several plasmid replication proteins. The RepA protein shares a 50 % identity in its 254 N-terminal domain, with the putative replication protein encoded in plasmid p2ABSDF from *Acinetobacter baumanii* SDF [32] and 38 % identity with the RepA protein of plasmid pMBO-1 from *M. bovis* Epp63 [20] (Fig. 2). Moreover, the sequence analysis of the proteins mentioned above revealed the presence of the N-terminal leucine zipper motif. A possible promoter for this putative RepA protein of the pMbo4.6 was identified at position _2668_TTTACA–17nt–TATAAT_2696_. Upstream of ORF3, a 320-bp intergenic region constituting the putative origin of replication that contained an AT-rich region followed by four direct repeats (DR), two 21-bp and two 22-bp, was identified (Fig. 1b). This set of repeated sequences resemble iteron structures and may play an important role in the control of plasmid replication. The direct repeats region showed high homology to the recently sequenced origins of replications of *Acinetobacter* plasmids (Fig. 1c). Inverted repeats (IR) and hairpin-loop structures which might confer regulatory effects on the replication of the plasmid were also noted. Analysis of this DNA fragment revealed the presence of a putative integration host factor (IHF) binding site, with high identity to the *E. coli* IHF consensus sequence [13], upstream of the first DR sequence (Fig. 1b). However, we did not detect sequences resembling the DnaA box. We also found that two other *M. bovis* strains T412 and IBA, coming from different geographic origins, possess plasmid similar to pMbo4.6, containing an identical *oriV* region (data not shown).

ORF4 encodes a 101-amino acid protein with 40 % identity with hypothetical protein DUF497 found in many unrelated bacteria (BLAST). All these molecules feature the COG2929 domain, which is highly conserved in bacteria, despite there being no functions described so far for members of this family.

### Binding of the RepA to *oriV* Fragments

**Fig. 3:**
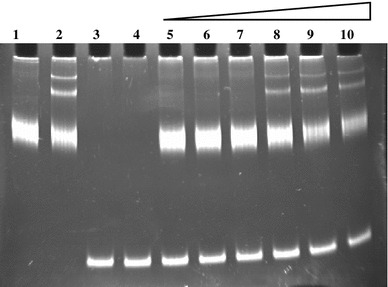
Specific binding of RepA protein to the replication origin. Gel mobility shift assay using 90 ng of orirep35 DNA fragment (378 bp), 109 ng of competitor DNA (94 bp) and increasing amounts of purified RepA protein (50–800 ng per binding reactions) (*lanes 5–10*). *Lanes 1* (orirep35) and *3* (competitor DNA) are negative controls without RepA protein, *lanes 2* (orirep35) and *4* (competitor DNA) contain 650 ng of RepA protein

To undertake pMbo4.6 RepA:DNA binding studies, the RepA protein was overproduced in *E. coli* BL21(DE3) cells. The RepA protein was purified by ion-exchange chromatography. SDS-PAGE revealed a clear band corresponding to a polypeptide of relative molecular weight about 45 kDa as expected from the amino acid sequence. As the DNA target, we used fragments containing repeated sequence motifs present throughout the putative *oriV* region (Fig. 1b). The most striking is the presence of direct repeats which occur four times. The 7-bp inverted repeat sequences designated as IR were found within DR and *repA* promoter regions. The interaction between Rep and iterons is known to be crucial for the regulation of plasmid replication. For this reason, we further confirmed the binding of RepA to the *oriV* region by the electrophoretic mobility shift assay (EMSA). Because of the variety and number of repeats scattered over a relatively long stretch of the *oriV* region, a number of overlapping fragments were amplified and tested for their ability to bind RepA. First, the whole pMbo4.6 replication region (Fig. 1b) was amplified by PCR using primers orirep3 and orirep5, yielding a 378-bp DNA probe designated orirep35. EMSA were conducted with orirep35 DNA and purified RepA (Fig. 3). The RepA protein was found to bind in vitro to orirep35 DNA and the amount of the probe which was retarded appeared to be proportional to the amount of RepA added to the binding reactions. The binding of RepA was specific since the formation of the RepA-iteron was unaffected by competition with unrelated DNA. To further assess *oriV* functionality, EMSA were performed using PCR-generated short segments of *oriV*: the orirep 32 probe (184 bp) containing an AT-rich region and the orirep15 probe (186 bp) comprising four direct repeats (Materials and methods). As expected, the RepA shifted only the iteron-containing part of the *oriV* (data not shown).

### RepA-Mediated Transcriptional Autoregulation

**Fig. 4:**
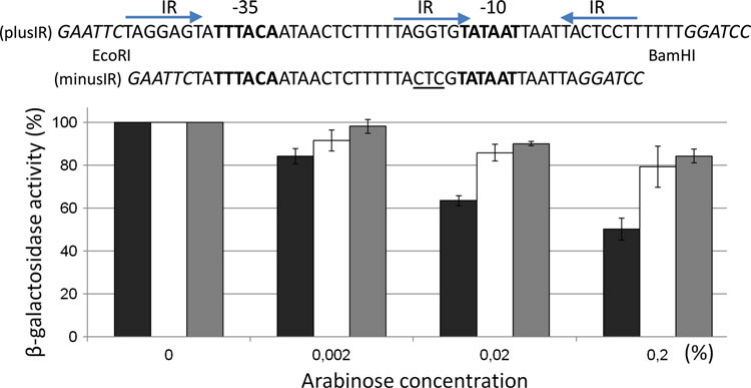
Autorepression of the *repA* promoter. Cultures of the MC1061 strain contained derivatives of plasmid pRS415 bearing the *lacZ* ^+^ reporter gene in transcriptional fusion with a minimal WT *repA* promoter fragment (pRSplusIR, *black*), or a mutated promoter (pRSminusIR, *white*) or an unspecific promoter of *ecoVIIIM* DNA methyltransferases (*gray*). β-Galactosidase activity in exponentially growing cells harboring appropriate plasmids was measured in the absence or presence of the RepA protein (pACYCaraRep), induced by a different L-arabinose concentration by 1 h. The values obtained in the absence of induction were assumed to be 100 %. All values are mean ± SD for at least three independent experiments

Analysis of the nucleotide sequence of the region located upstream of the *repA* gene allowed us to distinguish a putative promoter located 70-bp upstream of the start codon of *repA* (Fig. 1b). A double stranded synthetic oligonucleotide containing the promoter region of the *repA* gene was prepared by annealing megaD and megaG oligonucleotides (Table 1) and then inserted upstream of the promoter-less reporter *lacZ* gene between Eco*RI* and Bam*HI* sites in pRS415. The resulting plasmid pRSplusIR (Fig. 4) was introduced into *E. coli* MC1061 cells containing resident plasmid pACYCaraRep and then β-galactosidase activity was assayed to examine promoter strength. The obtained results confirmed the presence of a strong promoter within the cloned fragment (13,094 ± 1391 Miller units). A consistent decline of β-galactosidase activity value was observed after L-arabinose induction of the *repA* gene expression (Fig. 4). We also checked the level of RepA-mediated repression of expression *repA* gene in cells containing the promoter region without any IR sequences carried by pRSminusIR plasmid (Fig. 4 13,753 ± 437 Miller units). In this case, there was no significant decrease in the RepA-dependent β-galactosidase activity level. Similarly, lack of a RepA-inhibition effect was observed for the unspecific promoter of the *ecoVIIIM* methyltransferases gene [25]. Also, we noticed a lower culture growth rate after arabinose addition (data not shown). This effect was observed earlier both for *ara* mutants (MC1061) which non-metabolize l-arabinose [6] and in the case of the induction of expression of genes from a strong promoter (i.e., *ompA* expressed from *P*_araBAD_) [14].

## Discussion

Plasmids have played a major role in the ability of bacteria to adapt to specific environmental changes. Most isolates of *M. bovis* contain multiple plasmids although our knowledge concerning their functions remains limited. We discovered that the *M. bovis* strain ATCC 10900 harbors two plasmids of different sizes. The larger yet uncharacterized, approximately 20 kb, was designated pMbo20, and the much smaller plasmid was sized at 4658 bp.

The data obtained from the sequence analysis suggest the presence of two modules responsible for the replication and mobilization for conjugative transfer in pMbo4.6. Such modules are commonly found in different combinations within many small plasmids residing in Gram-negative bacteria such as neisseriae [33] and *Serratia marcescens* ACE2 [21]. These modules also occur in some plasmids from Gram-positive bacteria: *Paracoccus pantotrophus* DSM 11072 [2], *Enterococcus faecalis* [22], and *Pseudoalteromonas* sp. 643A [8].

Mobilizable plasmids usually carry a mobilization gene (*mob*) encoding a specific relaxase and the origin of transfer (*oriT*) from which the initiation of conjugative transfer occurs [11]. A key role in this process is played by Mob proteins (relaxases) which mediate cleavage of the phosphodiester bond within the *oriT* sequence crucial for initiation of DNA transfer in both conjugative and mobilizable plasmids. Functionally, MobA proteins act by binding and nicking double stranded DNA at the origin of transfer (*oriT*) site ready for DNA transfer, and are responsible for re-ligating the cleaved DNA strands once the transfer of DNA is achieved. Plasmid pMbo4.6 encodes the mobilization function; however, no typical *oriT* consensus sequence could be found which would place it in any of the oriT-relaxase systems [12].

Rep-type proteins bind to tandem arrangements of directly repeated sequences to establish the Rep-iteron initiation nucleoprotein complex. The Rep protein of some replicons, such as pPS10 and pSC101, have an important second function; they recognize inversely repeated sequences (operators) which overlap the promoter of their own genes, acting as self-repressors [9]. This is the case in our study. IRs overlapping the *repA* promoter gene show a high degree of homology to those present in the iterons region. However, the RepA-dependent degree of repression of *P*_*repA-lacZ*_ transcription obtained in our conditions was lower (50 %) than in the case of other known RepA proteins published elsewhere. We believe it is possible that the level of repression depends on the number of IR sequences present in cloned DNA fragments used in our work, similar to the results obtained for RepA from pPS10 [10]. They have shown that depending on the IRs context, the level of inhibition on the *P*_*repA-lacZ*_ fusions changed from 30 to 88 % under RepA delivery.

The essential components of the pMbo4.6 replication region were shown to be contained within a 0.3-kb DNA segment. The identified loci has a number of features commonly found in iteron-containing plasmids including the *repA* transcriptional promoter (*P*_*repA*_) and an AT-rich sequence usually associated with the strand melting of the origin. Four binding sites for the RepA protein are also found centrally within the intergenic region. The location of the RepA binding sites upstream of the *repA* gene appears to be a common feature among this family of replicons. The number, length, and arrangement of these were not only found to be variable but also function as binding sites for their respective Rep proteins. Analyzing the pMbo4.6 replication origin, we found significant homology of this region to several sequences found in *Acinetobacter* plasmids. Each replicon contained four direct and perfectly conserved repeats and the replicase gene, suggesting a similar mechanism of replication. These plasmids have been isolated from different genera, however, as proposed by Rossau [26] and co-workers, because *Moraxella* and *Acinetobacter* are phenotypically and genotypically related they have been classified as members of the family Moraxellaceae. The presence of significantly similar sequences in different bacteria may suggest the plasmids flow through a horizontal gene transfer, phenomenon spread among members of the *Acinetobacter* genus [3]. We also noticed that the RepA proteins of pMbo4.6 and p2ABSDF share significant similarity. Both replicase proteins belong to the Rep-3 superfamily so it may be hypothesized that their coding sequences derive from a common ancestor. However, both plasmids were isolated from bacteria found in different and geographically separated locations which suggests that the *mob* genes present in both plasmids might be involved in effective genetic transfer. It is worth mentioning that both genera may easily acquisite genetic information through natural transformation, contributing significantly to bacterial adaptation in a novel environment.

Several other types of direct repeats of unknown function are also present in the region immediately upstream of the pMbo4.6 iterated region. For example, the sequence 5′-AACAAGAAAA-3′ is repeated twice upstream of the AT reach region

Altogether, our results suggest that pMbo4.6 replicon belongs to theta replicating plasmids. The sequence analysis results revealed *mobC* and *mobA* genes as well as an inverted repeat between them suggesting that the pMbo4.6 plasmid might be mobilized in the presence of conjugative plasmids. However, further research on the characteristics of *M. bovis* ATCC 10900 plasmids is needed.
